# Smartphones, Emotions and Bullying Among Adolescents: A PRISMA Systematic Review

**DOI:** 10.3390/jintelligence14070131

**Published:** 2026-07-01

**Authors:** Carolina Bello-Correas, Teresa Alzás, Laura Alonso-Díaz

**Affiliations:** 1 International Doctoral School, University of Extremadura, 10071 Cáceres, Spain; cbelloco@alumnos.unex.es; 2 Department of Sociology, University of Extremadura, 10071 Cáceres, Spain; teresaag@unex.es; 3 Department of Education, University of Extremadura, 10071 Cáceres, Spain

**Keywords:** emotional education, emotional intelligence, cyberbullying, adolescence, secondary education, information and communication technologies

## Abstract

This systematic review, following PRISMA 2020 guidelines, analyzes scientific literature on bullying and cyberbullying among adolescents (aged 12–16) in educational settings, focusing on ICT, smartphone hyperconnectivity, and emotional education. An exhaustive search across Web of Science, Scopus, PubMed, Dialnet, CSIC, SciELO, and Google Scholar identified 34 empirical studies. A narrative synthesis was performed due to methodological heterogeneity. The synthesized evidence suggests that cyberbullying frequently acts as a persistent extension of school violence, where continuous digital exposure makes it difficult for victims to emotionally disconnect. Empirical data indicate a concerning correlation between prolonged bullying and psychosocial distress, including self-harming behaviors and suicidal ideation. Furthermore, results highlight systemic gaps: heightened vulnerability is reported among girls and LGBTQ+ students, alongside disparities between public and state-subsidized schools regarding institutional involvement and emotional support resources. Educational implications suggest reactive protocols are insufficient. Evidence supports that systematic emotional education, enhancing socio-emotional skills like impulse control, empathy, self-esteem and emotional regulation, acts as a key protective factor. Consequently, fostering “digital emotional intelligence” emerges as a promising preventive educational strategy to protect adolescents’ well-being in hyperconnected environments.

## 1. Introduction

### 1.1. International Regulatory Landscape and Digital Well-Being in Adolescence

There is growing concern in the international community about the harmful consequences of the inappropriate use of mobile devices and social media in adolescence (Winstone et al., 2024). This reality is leading to varying measures being taken at the political level in different countries. The most forceful of these is in Australia, which on 10 December 2025 passed a pioneering law prohibiting minors under the age of 16 from maintaining active social media accounts to protect their digital well-being and mental health, and which is being observed by other governments as a possible regulatory model. Meanwhile, in the United States, legislative initiatives aimed at removing age restrictions on access to digital platforms have been debated, generating controversy over the role of the state in protecting minors (Anderson et al., 2023).

In Europe, regulatory efforts to protect minors in the digital environment have intensified. The Digital Services Regulation (European Parliament and Council of the European Union, 2022; European Commission, 2022) establishes obligations to improve the safety and well-being of minors on digital platforms. At the same time, the European Parliament has proposed a minimum age of 16 for accessing social media, allowing use from the age of 13 with parental consent (European Parliament, 2025). In January 2026, the European Commission (2026) continues to evaluate this measure, and several Member States are testing enhanced age verification systems. In this context, France has passed a bill in the National Assembly prohibiting access to social media for children under 15, which will come into force in September 2026 (Assemblée nationale, 2025), highlighting the need for comprehensive preventive approaches that combine regulation, digital skills and emotional education to reduce digital violence in adolescence. These measures invite reflection on the increasing use of mobile devices and access to ICTs among teenagers.

### 1.2. Patterns of Violence in Digital Environments

One of the most notable aspects is how bullying has created new hazards in both face-to-face and digital environments, giving rise to cyberbullying and other forms of online victimisation (Livingstone et al., 2022). The present article considers digital bullying or cyberbullying as a form of violence that takes place in virtual environments, using ICT (instant messaging and social networks). As with traditional bullying, it involves insults, threats and humiliation, and uses the digital environment to distribute violent, intimidating and humiliating content. Practices such as harassment, spreading lies (trolling), frapping (impersonation), catfishing (creating fake profiles) and doxing (spreading personal data) are used, in addition to spreading rumours and circulating humiliating and degrading photos and videos (dissing) (Salawu et al., 2020). It is a form of violence characterised by continuous aggression towards a person through technological means (Cortés-Alfaro, 2020). Both bullying and cyberbullying cause significant psychosocial and emotional damage to victims, manifesting itself in the form of anxiety, fear, irritability, sleep disorders, somatisation and, when the suffering is prolonged, suicidal thoughts (Gini & Pozzoli, 2009; Kowalski et al., 2014; Van Geel et al., 2014). The increase of digital bullying is related to the expansion of the use of social networks and digital platforms in adolescence, as well as to dynamics of hyperconnectivity and continuous online exposure (Livingstone et al., 2022; UNICEF, 2024).

According to the National Cybersecurity Institute (INCIBE, 2020), cyberbullying occurs because the perpetrator perceives it as a joke, is seeking revenge for a previous dispute, or derives pleasure from embarrassing the victim to feel superior. Aggressors often have low self-esteem and use digital violence to gain a sense of dominance, or because of social pressure (Zych et al., 2015, 2018). Vulnerable victims are usually those with racial, cultural, sexual, or physical differences, and they have low self-esteem, emotional insecurity, and a lack of social support, which makes them more exposed to harassment and consequently easy targets (Livingstone et al., 2022).

Cyberbullying usually manifests itself indirectly, as it does not require a physical encounter between the aggressor and the victim, unless it stems from a previous face-to-face aggression. The aggressor often acts anonymously, which facilitates virtual violence and generates feelings of vulnerability and helplessness in victims who are unable to identify the aggressor. Furthermore, cyberbullying extends its reach beyond the school environment, invading the victim’s privacy at home and creating perceptions of legal helplessness (Waasdorp & Bradshaw, 2015). Even if the victim reports or blocks the aggressor, harassment can continue with new accounts and the dissemination of shared images.

The absence of direct contact in cyberbullying makes it difficult for the aggressor to develop empathy towards the victim, and in some cases, there is a reversal of roles when the perpetrator becomes the victim through acts of revenge. The consequences of cyberbullying affect not only the victims, but also the aggressors, their families and other Internet users. Victims may experience low self-esteem, anxiety, depression, social isolation, and even suicidal thoughts (González-Cabrera et al., 2018). In addition, witnesses may experience consequences by feeling complicit in the violence or fearing that they will become future victims (Bastiaensens et al., 2014).

Sociodemographic factors are pivotal in understanding the distribution and nature of school violence. Current evidence indicates that aggressive behaviors are significantly more prevalent in secondary education settings (Domínguez-Mora et al., 2019). The manifestation of this violence tends to evolve according to the developmental stage: physical aggression is more frequent among adolescents aged 11 to 14, while psychological and digital forms of violence become more prominent from the age of 15 onwards (Smith et al., 2019). Regarding gender disparities, research shows that males are more frequently involved in direct physical aggression and report higher rates of cyber-perpetration, whereas females are more likely to experience relational victimization and indirect forms of digital harassment (Valenzuela-Aparicio et al., 2023; Waasdorp & Bradshaw, 2015). Furthermore, the institutional framework plays a decisive role; however, these differences must be contextualized. For instance, evidence from the Peruvian context indicates that students in public secondary schools perceive greater barriers to accessing reporting systems compared to private institutions (Zegarra Chapoñan et al., 2022). Similarly, studies conducted in Mexico (Sandoval Martínez et al., 2022) and Spain (Giménez-Gualdo et al., 2018) suggest that state-subsidized and private schools often display different patterns of institutional involvement and offer more specialized emotional support resources than public centers. Consequently, these institutional differences highlight how the school’s specific socioeconomic and regional context can mitigate or exacerbate digital peer dynamics and should not be interpreted as universal trends.

### 1.3. Affective Processes and Digital Emotional Intelligence: From Impulse Control to Emotional Regulation

In short, the increase in cyberbullying among adolescents is generating growing social concern, especially in schools. Although violence in educational contexts is not a new phenomenon (Zych et al., 2015), the expansion of social media and digital environments has transformed its scope, intensity and persistence. Recent evidence indicates that uncontrolled use of digital platforms and social media has a negative impact on adolescents’ emotional management and can encourage the normalisation and amplification of violent behaviour among peers, increasing psychological distress and associated psychosocial risks (UNESCO, 2023; UNICEF, 2024; OECD, 2026). Furthermore, when violence extends to the virtual environment, the harm invades the home, a space that should be a safe environment for minors, intensifying emotional distress and the risk of suicidal ideation in the face of perceived helplessness (González-Cabrera et al., 2018; Modecki et al., 2014). Consequently, this landscape demands transitioning toward “digital emotional intelligence”. Rather than an established empirical mechanism, this concept represents an emerging educational framework that must be distinguished from related constructs. While conventional emotional intelligence relies on face-to-face non-verbal cues, digital emotional intelligence specifically addresses the affective demands of virtual environments, such as screen-induced hyperstimulation and online anonymity. Furthermore, it differs from basic digital literacy—focused on technical ICT skills—and from general emotional regulation, which functions as a specific mechanism within this broader framework (Audrin & Audrin, 2023).

This necessity arises because digital platforms, designed for immediate gratification, generate a cycle of hyperstimulation that prioritizes impulsive reactions over reflective processing (Winstone et al., 2024). Such environments trigger high states of affective arousal which, combined with perceived anonymity and the absence of physical social cues, facilitates the online disinhibition effect and compromises impulse control mechanisms (Kowalski et al., 2014). Within this framework, emotional regulation serves as a vital mediating factor that provides the cognitive distance necessary to manage heightened arousal and resist the urge for digital retaliation (Zych et al., 2018; Winstone et al., 2024). Therefore, a robust digital emotional intelligence acts as an essential protective shield, mitigating the impact of hyperconnectivity and preventing emotional distress from manifesting as cyber-perpetration (Fragoso-Luzuriaga & Ramírez-Santiago, 2022; WHO Regional Office for Europe, 2024).

This study reveals that contemporary cyber-violence is driven by high affective arousal and failed impulse control in hyperconnected environments. Consequently, we provide a specific roadmap for school intervention: shifting from technical restrictions to fostering digital emotional intelligence as a key protective factor. These findings offer immediate evidence to standardize protocols and bridge the resource gap between public and private institutions through targeted, emotionally grounded prevention. The study answers the following research questions:

- (RQ1) What are the main study methodologies used in research on bullying and cyberbullying among adolescents?
- (RQ2) What are the demographic patterns (e.g., gender, orientation) of victimization and perpetration?
- (RQ3) What is the role of ICT and mobile devices in these dynamics?
- (RQ4) How do emotions and their management influence the context of bullying and cyberbullying?

By synthesizing evidence, this review provides a strategic framework to transform school interventions into effective, emotionally-grounded prevention strategies that address the immediate needs of hyperconnected adolescents.

## 2. Materials and Methods

### 2.1. Study Design

The present work was conceived as a systematic review of the literature following the guidelines of the PRISMA 2020 statement, with the aim of ensuring the transparency, comprehensiveness, and reproducibility of the process of identifying, selecting, evaluating, and synthesising the included studies. The review focuses on empirical research on bullying and cyberbullying in secondary school adolescents, considering both face-to-face and digital manifestations. This systematic review was conducted and reported in accordance with the PRISMA 2020 statement. The completed PRISMA 2020 checklist (Page et al., 2021a) is provided as Supplementary Material S1.

The methodological process includes: (a) defining the general objective, and four research questions on methodologies, sociodemographic characteristics, ICT use and the role of emotions; (b) selecting specialised databases and search engines; (c) establishing explicit inclusion and exclusion criteria; (d) identifying and screening records; (e) evaluating the eligibility of studies; and (f) narratively synthesising the results obtained.

The review followed a sequential procedure in several stages. First, a systematic search was conducted in the Web of Science, Dialnet, CSIC, and SciELO databases, supplemented by Google Scholar, to ensure broad coverage of recent scientific literature. In response to peer review recommendations, the search strategy was subsequently reinforced through a bibliographic update in Scopus and PubMed to enhance the methodological rigor, traceability, and reproducibility of the review. Subsequently, filters were applied relating to the publication period (2018–2023). This specific timeframe was selected to analyze the most recent and consolidated scientific production on bullying and cyberbullying among adolescents within a homogeneous five-year interval. Since the review commenced in 2024, inclusion of the ongoing year was avoided to prevent the bias of partial data or publications still in the process of indexing. Additional filters included the study population (adolescents aged 12 to 16), the type of document (scientific articles), the language (Spanish and English), and the thematic content linked to school bullying or cyberbullying. Duplicates were then removed, and titles, abstracts, and full texts were reviewed according to the established eligibility criteria. Finally, the selected studies were organised through a narrative synthesis into predefined thematic categories.

The entire process of identifying, screening, excluding and selecting studies is shown in the PRISMA flow diagram, which reflects the transition from the initial 172 records to the final inclusion of 34 articles for analysis. This methodological design provided an up-to-date, consistent and well-founded overview of bullying and cyberbullying in adolescence during the period 2018–2023.

In alignment with the objectives of the review, the search strategy was not exclusively restricted to emotional descriptors. This intentional decision addressed the necessity of obtaining a broader, holistic understanding of bullying and cyberbullying during adolescence, integrating social, technological, educational, familial, and emotional dimensions. Consequently, emotions and emotional regulation were analyzed as transversal factors within the general phenomenon rather than isolated variables. Following this approach, the review was structured using an adapted PICO framework suitable for exposure-based observational research: P (Population): adolescents aged 12 to 16 enrolled in compulsory secondary education; I/E (Intervention/Exposure): use of ICT, mobile devices, social networks, and contexts of bullying and cyberbullying; C (Comparison): differences based on gender, sexual orientation, type of educational institution, and involved roles; and O (Outcomes): psychosocial, emotional, and educational consequences, including self-esteem, anxiety, emotional regulation, coping strategies, and mental health.

### 2.2. Eligibility Criteria

To ensure the relevance, consistency and methodological quality of the studies included in this systematic review, explicit eligibility and exclusion criteria were established prior to the selection process (Table 1).

These eligibility and exclusion criteria enabled the development of a rigorous selection process aligned with the objectives of the review, facilitating a comprehensive and integrated analysis of bullying and cyberbullying among adolescents during the 2018–2023 period.

### 2.3. Sources of Information and Search Strategy

Relevant studies were identified through a systematic and exhaustive search of various databases and academic search engines relevant to the fields of social sciences, educational psychology and adolescent research, as well as for the diversity of articles they index.

The following were consulted: Web of Science (WOS); Dialnet; CSIC (Spanish National Research Council); Scielo; and Google Scholar, used as a complementary search engine to broaden the sensitivity of the process. The initial search was completed on 15 September 2024. Subsequently, in response to the recommendations derived from the peer-review process, a bibliographic update was conducted in Scopus (Q1 & Q2) and PubMed. This update allowed for the incorporation of studies published in indexed, internationally recognized scientific journals (final search completed on 10 May 2026).

Regarding descriptors and Boolean operators, the search combined descriptors in Spanish and English to identify national and international scientific literature, using Boolean operators (AND/OR) and grouping terms to optimise the retrieval of potentially eligible studies. In Spanish, the following descriptors were used: “Adolescencia”, “Secundaria”, “Acoso escolar”, “Acoso virtual”, “ciberacoso”, applying the search formula “Adolescencia OR Secundaria AND Acoso escolar OR Acoso virtual OR ciberacoso”. In English, the following descriptors were used: “adolescents”; “secondary school”; “bullying”; and “virtual bullying”, using the equation “Adolescence OR Secondary School AND School Bullying OR Virtual Bullying OR virtual bullying OR Cyberbullying”. The search equations were adapted to each database according to its specific criteria (title, abstract, all fields or journal indexes), maintaining semantic consistency between terms and operators (Table 2).

The combined search across all information sources yielded 3018 records. This total corresponded to the sum of records identified in Scopus (*n* = 873), PubMed (*n* = 1973), Web of Science (*n* = 18), Dialnet (*n* = 42), SciELO (*n* = 15), CSIC (*n* = 21), and Google Scholar (*n* = 76), prior to duplicate removal and eligibility screening.

The research process was carried out in several stages, including the identification of records in scientific databases, the removal of duplicates, the screening of titles and abstracts, the review of full texts, and the final eligibility assessment. Following the application of inclusion and exclusion criteria, those studies considered methodologically and thematically relevant for the final review were selected.

The bibliographic update conducted in Scopus and PubMed strengthened the methodological rigor, reproducibility, and international scope of the review by incorporating recent research published in high impact (Q1 and Q2) indexed scientific journals. This update allowed for the addition of five new empirical studies related to bullying, cyberbullying, emotional well-being, mental health, and digital behaviors in adolescents, supplementing the original systematic review.

**Figure 1:**
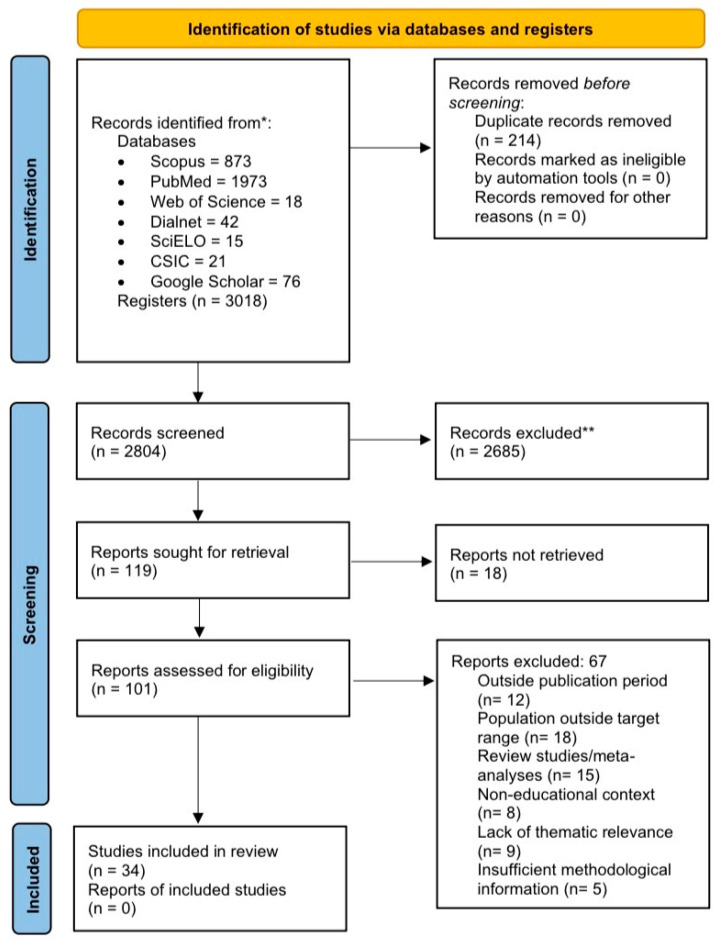
PRISMA Flow Diagram. * Number of records identified from each database or register searched. ** Records were excluded by human and automation tools.

After the complete screening and eligibility assessment process, the final sample consisted of 34 studies included in the systematic review. The detailed process of identification, selection, eligibility assessment, and final inclusion of the studies is presented in the PRISMA 2020 flow diagram (Figure 1).

### 2.4. Study Selection and Synthesis Process

The study selection process was carried out following the recommendations of the PRISMA 2020 statement, with the aim of ensuring a transparent, reproducible, and systematic procedure. This process consisted of three main phases: identification, screening and eligibility assessment, concluding with the final inclusion of studies that met all the predefined criteria. At the same time, a narrative synthesis strategy was applied to integrate the findings of the selected studies.

The bibliographic update executed in Scopus and PubMed enabled the incorporation of recent studies published in high-impact, internationally indexed scientific journals, thereby reinforcing the methodological consistency and scientific scope of the review. Finally, the definitive sample comprised 34 empirical studies focusing on bullying and cyberbullying among adolescents enrolled in compulsory secondary education.

Once the eligible studies were selected, they were analysed and synthesised. Given the considerable heterogeneity of the designs, variables, approaches and measurement methods, the possibility of conducting a quantitative meta-analysis was ruled out. Instead, a narrative synthesis was used, structured around the research questions and previously defined thematic categories:

- Methodologies used in the study of school bullying and cyberbullying.
- Sociodemographic characteristics of the adolescents involved.
- Role of ICT and mobile devices in the dynamics of bullying.
- Relevance of emotions, self-esteem, and socio-emotional skills.

The synthesis was applied through a process of constant comparison, integrating the findings of the studies and standardising the terminology to promote interpretative consistency. This approach made it possible to identify common patterns, divergences and knowledge gaps, offering a structured overview of the current state of research between 2018 and 2023.

### 2.5. Narrative Assessment of Methodological Limitations

A Narrative assessment of methodological limitations was carried out with the aim of evaluating the methodological quality of the included studies and determining the extent to which their limitations could influence the validity of the results. Given that the 34 selected studies present heterogeneous methodological approaches—mainly quantitative, but also qualitative and mixed—a detailed assessment of key aspects related to study design, participant selection, variable measurement, and data analysis was carried out, following the general recommendations established in PRISMA 2020.

In selection, most studies used non-probability samples (e.g., convenience samples in educational centres), which limits generalisation, although they adequately described the population, age, and inclusion criteria (Higgins et al., 2024). In terms of information, the use of validated instruments (e.g., EBIP-Q, ECIP-Q, SDQ, Rosenberg) predominated, reducing measurement error; however, there still exists a risk of self-reporting, especially in sensitive variables such as victimisation, suicidal ideation, or aggression (Van de Mortel, 2008; Gnambs & Kaspar, 2024). In terms of confounding, several studies incorporated sociodemographic covariates (gender, age, socioeconomic status, family context) but did not always report systematic control strategies (Sterne et al., 2016). Regarding design and reporting, quantitative studies showed good methodological transparency; in qualitative studies, the description of the analysis and saturation was more irregular, with a moderate risk to credibility and transferability (CASP, 2018). Finally, although no formal tests were applied, the existence of publication bias towards significant results or high prevalences was considered plausible (Page et al., 2021b).

### 2.6. Synthesis Method

Given the notable methodological heterogeneity present in the studies, both in design (quantitative, qualitative, and mixed) and in the variables evaluated, the instruments used, and the educational contexts analysed, it was not possible to perform a quantitative meta-analysis. Consequently, we decided to use narrative synthesis, following the recommendations of the PRISMA 2020 statement for reviews without statistical aggregation.

The synthesis integrated the findings of the included studies according to the research questions and previously defined thematic categories (Table 3): (a) methodologies used in the study of school bullying and cyberbullying; (b) sociodemographic characteristics associated with victimisation and perpetration; (c) use of information technologies and mobile devices in bullying dynamics; and (d) role of emotions, self-esteem, and emotional intelligence in the experience of bullying.

Relevant information on all 34 studies was extracted relating to design, sample, instruments, variables analysed, and main results. Although the studies used different terminology, conceptual standardisation was carried out—especially in terms such as “digital bullying,” “cyberbullying,” “problematic mobile phone use,” and “social-emotional skills” to facilitate cross-sectional comparison between studies.

The synthesis was carried out in four steps: (1) classification of the studies by analytical categories linked to the research questions; (2) systematic comparison within each category to detect convergences, divergences, and contradictions, taking into account the relationship between methodology and results; (3) identification of common patterns, such as the predominance of quantitative cross-sectional designs, the use of validated instruments, and the greater vulnerability of certain groups (e.g., LGBTQ+ adolescents), with self-esteem and emotional intelligence as modulating factors; and (4) contextualised critical interpretation of the findings, considering methodological limitations and the risk of bias, in order to weigh the available evidence in a balanced manner.

Given that the synthesis was based on narrative rather than quantitative procedures, statistical heterogeneity was not explored and no sensitivity analyses were performed. However, the conceptual heterogeneity present in variables, samples, and designs was recognised and integrated into the interpretation of the final results (Table 4). The use of a narrative synthesis provided a contextualised view of bullying and cyberbullying among adolescents, integrating the frequency and typology of behaviours with the role of the digital environment and the emotional factors and sociodemographic differences that shape young people’s experiences in school and virtual contexts.

## 3. Results

### 3.1. Methodologies Used in Research on Bullying and Cyberbullying Among Adolescents

The analysis of the 34 studies included shows a clear predominance of quantitative methodologies measuring prevalence, roles of victimisation and perpetration, and associated psychosocial variables (Table 5), the final 5 presented were introduced after peer reviewers’ suggestions.

Specifically, 29 studies adopted quantitative designs mainly descriptive, correlational, and cross-sectionals supported by validated psychometric instruments such as EBIP-Q, ECIP-Q, M-PPUS-A, EUPI-A, Rosenberg’s Self-Esteem Scale, and SDQ, as well as specific scales of digital aggression.

These instruments reflect an interest in quantifying both face-to-face and digital bullying and their relationship with variables such as self-esteem, depression, emotional intelligence, family attachment, and problematic mobile phone use, incorporating contextual variables (type of school, family environment, or socioeconomic status) in some cases.

Mixed methodologies were rare (four studies) and combined questionnaires with interviews or focus groups, providing greater interpretative depth, especially in research on emotional intelligence, grooming, homophobic bullying, and family dynamics. Only one qualitative study directly explored the emotional component of cyberbullying, addressing experiences such as shame, anxiety, and perception of institutional support, complementing the quantitative results (Table 6).

### 3.2. Sociodemographic Characteristics of Adolescents Who Suffer Bullying and Cyberbullying, and Associated Patterns of Victimisation and Perpetration

Studies agree that bullying and cyberbullying mainly affect adolescents aged 12 to 16, a developmental stage characterised by increased peer influence, identity construction and socio-emotional vulnerability (Páez-Esteban et al., 2020a, 2020b; Ruiz-Narezo et al., 2020; Zych et al., 2018). Consistent differences are observed in gender and diversity: girls have a higher prevalence of victimisation, especially in digital contexts, while boys show greater involvement in face-to-face and digital aggressive behaviours, associated in some cases with the pursuit of social status. LGBTQ+ adolescents are one of the most vulnerable groups, with higher rates of victimisation and psychological distress, especially when there are little family and social support (Rivera-Osorio & Arias-Gómez, 2020; Sánchez Aguilar et al., 2018).

In terms of patterns of involvement, the prevalence of victimisation ranges from 33% to 36%, perpetration from 14% to 22%, and more than 80% of adolescents identify themselves as observers of violence. The most common forms are verbal, physical, psychological/social, and cyberbullying (20–40%), showing continuity between in-person and digital bullying.

The synthesized evidence suggests the existence of disparities between public and state-subsidized schools regarding the application of intervention protocols. Specifically, state-subsidized institutions appear to report higher levels of institutional involvement, broader community engagement, and more comprehensive emotional support resources for students.

The family and school context play a significant associated role: low family cohesion, previous victimisation and low confidence in reporting channels are associated with higher risk and limited detection. Likewise, differences in emotional impact are observed, with higher suicidal ideation and low self-esteem in girls, externalising behaviours in boys and a particular emotional vulnerability in LGBTQ+ adolescents.

### 3.3. The Role of ICT and Mobile Devices in Bullying and Cyberbullying Among Adolescents

The studies analyzed consistently show that ICTs and mobile phones play a central role in peer bullying. Social networks and messaging platforms (especially WhatsApp, Instagram, and Facebook) are the main scenarios for cyberbullying, facilitating social exclusion, the aggressor’s anonymity, and the prolongation of harassment beyond the school environment. Behaviours such as insults, threats, identity theft, and the non-consensual dissemination of images are intensified by the absence of spatio-temporal limits. Furthermore, the emerging use of artificial intelligence-based tools in these contexts is increasing the persistence of the damage and its emotional impact (Fundación ANAR & Fundación Mutua Madrileña, 2025).

Intensive and problematic mobile phone use is significantly associated with greater exposure to digital risks, reduced self-control, and heightened vulnerability to victimization, especially among girls. Hyperconnectivity is directly linked to stress, compulsiveness, lower emotional well-being, and a greater likelihood of both suffering and perpetrating digital aggression.

Various forms of cyberbullying are documented (public humiliation, frapping, catfishing, dissemination of content without consent, and blackmail) facilitated by the speed of dissemination, the multiplicity of channels, and limited parental supervision. Family support and emotional and cybersecurity education emerge as protective factors, while the limited use of institutional reporting systems hinders early detection and encourages the persistence of digital harassment.

### 3.4. Emotions, Bullying and Cyberbullying

Emotions are central to understanding these phenomena. Thirteen of the 34 reviewed studies confirm that variables such as shame, anxiety, depression, self-esteem, and emotional clarity have a decisive influence on vulnerability and psychosocial impact.

Victims experience high levels of anxiety, depression, suicidal ideation, and severe shame—particularly associated with public digital exposure. Notably, these impacts show a higher prevalence in girls and in situations of prolonged victimization, where the digital amplification of the damage encourages concealment and makes seeking help difficult.

Finally, aggressors typically display traits such as narcissism, low empathy, and difficulties with emotional regulation, which are facilitated by the anonymity and emotional distance of ICTs. Bystanders often experience guilt, fear, or helplessness, reinforcing their inaction. Consequently, literature highlights socio-emotional skills (emotional intelligence, empathy, resilience) as essential protective factors, whereas their deficit drastically increases vulnerability in digital environments (Table 7).

## 4. Discussion

While the impact of cyberbullying on adolescent well-being is well-documented, the present systematic review contributes to the discourse of this Special Issue by examining the protective and regulatory role of emotional intelligence and socio-emotional competence in digital peer dynamics. Specifically, the synthesized evidence corroborates that the hyperstimulation and immediate gratification inherent to smartphones and social networks are linked to high states of affective arousal among adolescents. In these hyperconnected environments, characterized by perceived anonymity and a lack of physical social feedback, traditional impulse control mechanisms are frequently compromised.

Therefore, our findings indicate that cyberbullying is not merely a conduct issue, but is frequently associated with deficits in emotional regulation and the manifestation of social intelligence within virtual spaces. When adolescents lack the capacity to process high emotional arousal, this distress is frequently related with externalized digital aggression or internalized symptoms, which are linked to a greater psychosocial vulnerability of victims. Consequently, psychological well-being in adolescence is inextricably linked to the capacity to navigate these complex digital environments. This highlights digital emotional intelligence not only as a necessary preventive tool against violence, but as an essential educational scaffold for the intellectual and personal development of emerging adolescents.

Therefore, the results of this systematic review confirm that bullying and cyberbullying in adolescence are complex and multidimensional phenomena, influenced by individual, social, emotional and technological factors. From an integrative approach, the findings corroborate trends described in recent literature and provide nuances for understanding the current dynamics of peer bullying in digitalised educational contexts.

The predominance of quantitative studies based on standardised instruments reflects the consolidation of a field focused on measuring prevalence and psychosocial correlations. However, the scarcity of qualitative and mixed designs limits our understanding of the subjective and relational dimensions of bullying, especially in digital environments, where the emotional impact is central (Higgins et al., 2024).

Sociodemographic differences confirm greater emotional impact on girls and high vulnerability in LGBTQ+ adolescents, while boys show greater involvement in aggressive behaviours, associated in some cases with the pursuit of social status, in line with differential socialisation processes (Valenzuela-Aparicio et al., 2023; UNESCO, 2023). These results underscore the need for diversity-sensitive educational interventions (Kosciw et al., 2024; OECD, 2026). Some studies also identify correlations between bullying victimization, sedentary behaviors, and lower participation in physical activities, particularly among adolescents experiencing emotional distress or situations of social exclusion (Pulido et al., 2019). Regarding ICT, cyberbullying is an extension of face-to-face bullying, expanding its temporal, spatial and emotional reach. Hyperconnectivity, problematic mobile phone use and limited adult supervision intensify exposure and make it difficult for victims to disconnect emotionally (Álvarez Menéndez & Moral Jiménez, 2020; Smahel et al., 2020; Livingstone et al., 2022). Recent studies also highlight the emergence of new digital dynamics associated with cyberbullying. Specifically, Chao et al. (2023) found that problematic TikTok use among adolescents correlated with higher levels of anxiety, depression, loneliness, emotional distress, and cybervictimization, thereby reinforcing the relationship between hyperconnectivity, problematic social media use, and psychosocial vulnerability.

Emotions emerge as a cross-cutting theme: shame, anxiety, depression and low self-esteem are common consequences of victimisation, with implications for long-term mental health (Fragoso-Luzuriaga & Ramírez-Santiago, 2022; Modecki et al., 2014; WHO Regional Office for Europe, 2024). In turn, low empathy and difficulties with emotional regulation are associated with perpetration, reinforcing the preventive value of social-emotional skills. Furthermore, recent research underlines the importance of positive childhood experiences, family connectedness, and supportive social environments as protective factors against bullying victimization and perpetration. Specifically, Crouch et al. (2023) observed that adolescents with higher levels of family resilience, emotional support, and positive interpersonal relationships exhibited lower involvement in school bullying dynamics.

Finally, significant challenges remain to be addressed, such as low confidence in institutional reporting systems, differences between public and private educational institutions in terms of coping strategies, and the limited inclusion of non-binary identities and trans adolescents in empirical research which restricts the design of fully inclusive and effective educational responses, resulting in the invisibility of the harm caused to this group.

## 5. Conclusions

The synthesis of the 34 included studies suggests that cyberbullying frequently acts as a persistent extension of face-to-face violence, amplifying public humiliation and the chronicity of harm. The synthesized literature suggests that smartphone hyperconnectivity can make it difficult for victims to emotionally disconnect. This continuous digital exposure is regularly associated with exacerbated psychosocial distress, particularly when adequate emotional support from family and educational environments is lacking, or when suffering is minimized and made invisible.

Crucially, the reviewed studies point to severe implications that might warrant increased collective awareness. Specifically, several studies seem to underscore a concerning correlation between prolonged bullying and self-harming behaviours or suicidal ideation—an issue that unfortunately remains treated as taboo in many educational settings. Furthermore, the synthesized evidence points to significant systemic gaps: the reported heightened vulnerability of girls and LGBTQ+ adolescents highlights the need for targeted, inclusive protection strategies. Similarly, regarding institutional responses, findings reveal disparities that should be more cautiously addressed as some studies suggest that, in specific socio-regional contexts, private schools frequently act more effectively and make cases of bullying more visible compared to public institutions. In any case, this highlights a critical need to standardize emotional support and reporting mechanisms across all educational centers.

The practical implications of these findings suggest that reactive policies and technological restrictions alone may be insufficient. Although literature consistently supports emotional education as a highly promising preventive strategy, the evidence indicates that its implementation remains insufficient and irregular in many educational contexts. In terms of prevention, the results support the systematic incorporation of emotional education as a tool to enhance socio-emotional skills—such as self-esteem, critical empathy, emotional regulation, and the construction of personal identity—which emerge as potential protective factors. Conversely, the absence of these skills appears closely associated with a greater vulnerability to both victimization and the perpetration of violent behavior. In this context, it is essential to develop educational and social policies that are firmly committed to emotional education, critical digital literacy, and the strengthening of intrapersonal factors. Preventing bullying and cyberbullying means going beyond reactive protocols to make a collective commitment to the ethics of care, the responsible use of technology, and the protection of children and adolescents’ emotional well-being as an unavoidable educational priority.

Finally, this review presents certain limitations. The review was not prospectively registered in PROSPERO or any other review registry, which may limit the transparency of protocol development and increase the risk of selective reporting. Nevertheless, the review process was conducted following PRISMA 2020 guidelines to ensure transparent reporting of the search, selection and synthesis procedures. Additionally, the predominance of quantitative cross-sectional studies limits causal inference and the understanding of the directionality between ICT use, socio-emotional distress, and school and digital bullying. The recurrent use of self-reports, though based on validated instruments, may introduce biases in particularly sensitive variables, such as victimization or suicidal ideation. Furthermore, the methodological heterogeneity of the included studies makes it difficult to directly compare prevalences, and a possible publication bias towards significant results cannot be ruled out. Despite these constraints, the synthesized evidence robustly supports the vital need to foster digital emotional education and intelligence in contemporary settings.

## Figures and Tables

**Table 1:** Inclusion and exclusion criteria.

Criterion	Inclusion Criteria	Exclusion Criteria
Type of publication	Empirical scientific articles	Reviews, meta-analyses, doctoral theses, bachelor’s and master’s theses
Population	Adolescents aged 12–16 in secondary education	University students, primary education, adults, non-educational populations
Publication period	Studies published between 2018 and 2023	Studies published outside this period
Language	English and Spanish	Other languages
Thematic content	Bullying and cyberbullying in adolescents	Studies unrelated to bullying/cyberbullying or focused on non-school violence
Study design	Quantitative, qualitative, and mixed methods studies	Non-empirical publications
Database screening	Unique eligible records	Duplicate records

**Table 2:** Database and search equations.

Database 1	Search Equations	Filters Applied
Web of Science	(“adolescents” OR “secondary education”) AND (“school bullying” OR cyberbullying)	2018–2023; articles; English/Spanish
Scopus	(adolescent OR adolescence OR adolescents OR “secondary education”) AND (bullying OR cyberbullying OR “school bullying”)	Articles; English/Spanish
PubMed	(adolescence OR adolescents OR “secondary education”) AND (“school bullying” OR cyberbullying OR “virtual bullying”)	Observational/comparative studies; 2018–2023
Dialnet	(adolescencia OR secundaria) AND (acoso escolar OR acoso virtual OR ciberacoso)	Scientific articles; Spanish
CSIC	(adolescencia OR secundaria) AND (acoso escolar OR ciberacoso)	Scientific articles; Spanish
SciELO	(adolescencia OR secundaria) AND (acoso escolar OR ciberacoso)	Scientific articles; Spanish
Google Scholar *	(“adolescents” OR adolescencia) AND (bullying OR cyberbullying OR ciberacoso)	Complementary search for additional relevant studies

* Google Scholar was used as a complementary search engine to broaden the sensitivity of the search process. To improve transparency and reproducibility, the first 200 records sorted by relevance were screened, from which 76 potentially relevant records were identified and entered into the selection process.

**Table 3:** Summary of the main findings of the review.

Category	Summary of Findings
Methodologies	Quantitative studies predominate (29), using validated questionnaires (EBIP-Q, ECIP-Q, SDQ, Rosenberg, TMMS-24, AF5, MPPUS-A). Four studies use mixed methodologies and one uses a qualitative methodology. Most designs are cross-sectional and correlational.
Type of bullying	Nine studies analyse face-to-face bullying, nine exclusively cyberbullying, and eleven both. The most common forms are verbal, social, and visual bullying, digital threats, and the dissemination of humiliating content.
Sociodemographic variables	Greater victimisation is observed in girls and LGBTQ+ adolescents. Boys show greater involvement in aggressive and cyberaggressive behaviour. The samples are concentrated in the 12–16 age group.
Emotions and mental health	Thirteen studies address emotions such as shame, anxiety, depression, low self-esteem, narcissism, dysphoria, and suicidal ideation. Shame emerges as a central emotion in cyberbullying, and low self-esteem as a predictor of vulnerability.
ICT and mobile phone use	The problematic use of mobile phones, unsupervised connection and risky behaviours (sexting, frapping, impersonation) are analysed. Intensive use of mobile phones and social networks predicts cyber victimisation and cyber aggression.
School and family context	Differences between public and private schools in coping strategies are identified. Secure family attachment acts as a protective factor, while family violence and offensive communication increase risk.
Coping strategies	Victims and observers resort to avoidance, disconnection, ignoring the aggressor, or seeking support. In public schools, aggressive or avoidant strategies predominate; in private schools, support and reporting predominate.
Consequences	Emotional problems (anxiety, depression, dysphoria), low self-esteem, behavioural problems and declining academic performance are evident. In cyberbullying, the damage is amplified by virality and anonymity.
Risk factors	LGBTQ+ identity, male gender (in aggression), substance use, family violence, offensive communication, narcissism, and problematic mobile phone use.
Protective factors	High self-esteem, emotional regulation, family supervision, positive school climate, and prosocial skills. Emotional education programmes have been shown to be effective in prevention.

**Table 4:** Thematic categories and variables analysed for the narrative synthesis of the included studies. Own elaboration.

Thematic Category	Description	Variables Included	Examples of Studies That Represent It
1. Methodologies used in the study of school bullying and cyberbullying	Analyses the methodological approaches used (quantitative, qualitative and mixed), as well as the instruments applied.	Type of design, analysis technique, instruments (EBIP-Q, ECIP-Q, SDQ, Rosenberg, etc.).	Cross-sectional quantitative studies on prevalence; mixed studies on grooming and emotions.
2. Sociodemographic characteristics and patterns of victimisation/perpetration	Examines how age, gender, identity, and sexual orientation influence bullying roles.	Age, gender, identity, sexual orientation, socioeconomic status, role (victim, aggressor, observer).	Studies on the greater vulnerability of LGBTQ+ adolescents; patterns of aggression and victimisation in the 12–16 age group.
3. Role of ICT and mobile devices in bullying	Explores the use of mobile phones, social media and digital platforms in the emergence and perpetuation of cyberbullying.	Type of ICT used, intensity of use, problematic use of mobile phones, social media, anonymity.	Studies on cyberbullying on WhatsApp and Instagram; problematic use associated with digital aggression.
4. Emotions, self-esteem and socio-emotional skills	Analyses the role of emotions and emotional skills in the experience of school and digital bullying.	Shame, anxiety, depression, self-esteem, narcissism, emotional intelligence.	Studies on digital shame, resilience, and low self-esteem as a vulnerability factor.
5. Coping strategies and responses to bullying	Addresses the responses of victims and their environment, comparing public and private centres and types of strategies.	Ignoring, asking for help, gathering evidence, aggressive reaction, family support, reporting.	Differences between public and private centres; low use of institutional reporting channels.

**Table 5:** Studies included in the review.

Authors & Year	Journal	Database	Study Focus
Álvarez Menéndez and Moral Jiménez (2020)	Health and Addictions/Salud y Drogas	Google Scholar	Problematic mobile phone use, social networks and self-control
Álvarez Quiroz et al. (2023)	Zona Próxima	WEB OF SCIENCE	Bullying, cyberbullying and self-esteem
Álvarez-Marín et al. (2022)	Revista de Psicodidáctica	WEB OF SCIENCE	Bullying and socio-emotional adjustment
Alvites Huamaní (2019)	Etic@net	WEB OF SCIENCE; DIALNET; ISOC_CSIC	Cyberbullying, depression and globalization
Artos et al. (2021)	Kronos	DIALNET	Cyberbullying and new technologies
Castro Castañeda et al. (2019)	IE Revista de Investigación Educativa	DIALNET	Risk variables associated with cyberbullying victimization
Cava et al. (2022)	Revista de Educación	WEB OF SCIENCE	Cybercontrol, dating violence and cyberbullying
Domínguez-Mora et al. (2019)	Acta Universitaria	DIALNET	Cyberbullying, psychological distress and suicidal ideation
Feijóo et al. (2021)	Psicothema	WEB OF SCIENCE; Scopus	Problematic internet use and cyberbullying roles
Fragoso-Luzuriaga and Ramírez-Santiago (2022)	RELATEC	CSIC	Emotional intelligence and grooming prevention
González-Calatayud and Prendes-Espinosa (2018)	Píxel-Bit	Google Scholar *	Cyberaggressors and secondary education
Herrera Hernández (2022)	Anuario de Derecho	Google Scholar *	Bullying and juvenile delinquency
Marín-Cortés (2020)	The Qualitative Report	WEB OF SCIENCE	Experiences of shame and cyberbullying
Martín-Criado and Casas (2019)	Aula Abierta	DIALNET	Peer-support intervention and bullying reduction
Martínez Sánchez et al. (2019)	Comunitania	DIALNET	Prevalence and intervention in school bullying
Carmona-Rojas et al. (2023).	Anales de Psicología	SciELO	Differences and similarities between bullying and cyberbullying
Páez-Esteban et al. (2020a)	Revista Cuidarte	WEB OF SCIENCE	Prevalence and associated factors of bullying
Páez-Esteban et al. (2020b)	Revista da Escola de Enfermagem	SciELO	Roles, violence types and determinants of bullying
Patiño-Masó et al. (2021)	Health and Addictions	PubMed	Alcohol consumption, health perception and family relationships in bullying
Rivera-Osorio and Arias-Gómez (2020)	Revista UIS Salud	SciELO	LGBT bullying and health implications
Romera et al. (2021)	Revista de Psicología y Educación	CSIC	Popularity, narcissism and aggressive behaviour
Ruiz-Narezo et al. (2020)	Bordón	WEB OF SCIENCE; Scopus	Victims, aggressors and violence cycles
Sánchez Aguilar et al. (2018)	Revista Sexología y Sociedad	Google Scholar *	Homophobic bullying
Sánchez-Domínguez et al. (2019)	EducateConciencia	DIALNET; Google Scholar *	Cyberbullying on social networks
Sandoval Martínez et al. (2022)	Diálogos sobre Educación	DIALNET	Coping strategies in cyberbullying
Urresti-Padrón et al. (2021)	Educatio Siglo XXI	WEB OF SCIENCE	Family attachment and bullying
Valenzuela-Aparicio et al. (2023)	Revista Electrónica Educare	WEB OF SCIENCE; Scopus; DIALNET	Emotional intelligence and bullying
Zegarra Chapoñan et al. (2022)	Revista Cubana de Enfermería	SciELO	Reporting systems and bystander intervention
Zych et al. (2018)	Revista de Psicodidáctica	WEB OF SCIENCE	Social and emotional competencies in bullying roles
García-Díaz et al. (2023)	Child Indicators Research	Scopus	Identification, testimony and reactions to bullying
Crouch et al. (2023)	Journal of School Health	Scopus	Positive childhood experiences and bullying
Chao et al. (2023)	Psychiatry Research	Scopus	TikTok use, psychosocial factors and bullying
Noll et al. (2022)	Nature Human Behaviour	Scopus	Internet behaviour after sexual abuse
Pulido et al. (2019)	Journal of School Health	Scopus	Bullying, physical activity and overweight youth

**Table 6:** Methodological distribution of the studies included.

Type of Study	Methodological Characteristics	Areas/Examples
Quantitative (29 studies)	Descriptive, explanatory, cross-sectional, longitudinal, correlational and case study approaches. Use of validated questionnaires (EBIP-Q, ECIP-Q, SDQ, Rosenberg, among others) and self-administered surveys.	Scope of bullying: in person, digital and both
Mixed-methods (4 studies)	Combination of questionnaires with interviews or discussion groups to deepen the understanding of emotional and relational dimensions.	Studies on emotional intelligence, grooming and coping strategies
Qualitative (1 study)	Semi-structured interviews and focus groups.	Digital sources of shame

**Table 7:** Emotions and their relationship with bullying and ICT.

Aspect Analysed	Summary of Results
The relevance of emotions	Thirteen articles highlight the central role of emotions in situations of bullying and cyberbullying.
Core emotions	Shame, anxiety, depression and low self-esteem.
Emotional factors	Emotional intelligence, self-concept and resilience. Studies include Las fuentes digitales de la vergüenza (Marín-Cortés, 2020) and Grooming e inteligencia emocional (Fragoso-Luzuriaga & Ramírez-Santiago, 2022).
Instruments used	Rosenberg Self-Esteem Scale, SDQ, and emotional competence questionnaires.
Relationship with ICT	Emotions mediated using social networks, as well as by risky practices such as sexting and grooming.

## Data Availability

Data is contained within the article or Supplementary Materials.

## Supplementary Materials

The following supporting information can be downloaded at: https://www.mdpi.com/article/10.3390/jintelligence14070131/s1, Supplementary Materials S1: PRISMA_2020_checklist.
